# Relativistic quantum key distribution system with one-way quantum communication

**DOI:** 10.1038/s41598-018-24533-6

**Published:** 2018-04-17

**Authors:** K. S. Kravtsov, I. V. Radchenko, S. P. Kulik, S. N. Molotkov

**Affiliations:** 1 Quantum Technology Centre of Moscow State University, Moscow, Russia; 2 A.M. Prokhorov General Physics Institute RAS, Moscow, Russia; 3 Faculty of Physics, Moscow State University, Moscow, Russia; 4 Academy of Cryptography, Moscow, Russia; 5 Institute of Solid State Physics, Chernogolovka, Moscow Rgn. Russia; 6 Faculty of Computational Mathematics and Cybernetics, Moscow State University, Moscow, Russia

## Abstract

Unambiguous state discrimination (USD) is one of the major obstacles for *practical* quantum key distribution (QKD). Often overlooked, it allows efficient eavesdropping in majority of practical systems, provided the overall channel loss is above a certain threshold. Thus, to remain secure all such systems must not only monitor the actual loss, but also possess a comprehensive information on the safe *‘loss vs. BER’* levels, which is often well beyond currently known security analyses. The more advanced the protocol the tougher it becomes to find and prove corresponding bounds. To get out of this vicious circle and solve the problem outright, we demonstrate a so called *relativistic* QKD system, which uses causality to become inherently immune to USD-based attacks. The system proves to be practical in metropolitan line-of-sight arrangements. At the same time it has a very basic structure that allows for a straightforward and comprehensive security analysis.

## Introduction

The workhorse of quantum cryptography has always been the BB84^1^ protocol, whose elegance mainly comes from the use of true single photons. It also enables quite unique and comprehensive security proof^2–4^. At the same time, no practical QKD protocol can use the same information carriers: they have to rely upon weak coherent pulses (WCPs) instead. As WCPs are formally infinite-dimensional quantum systems, there is always a non-zero probability of unambiguous discrimination of the transmitted states in the channel^5–7^. Thus, starting from some level of loss, conventional WCP-based QKD systems inevitably lose their guaranteed security. Corresponding thresholds are well-known for simple protocols as B92^8^ and WCP-based BB84^7^, but, to the best of our knowledge, still far from being found for popular WCP-based COW^9^ and DPS^10^ protocols, whose security proofs, thus, may be considered incomplete. To avoid this potential security breach at high channel loss we argue that additional measures have to be taken in the protocol design to completely disallow masking unsuccessful unambiguous state discrimination (USD) in losses.

Earlier, many efforts were directed towards developing protection against a much more narrow than USD type of attack, the photon number splitting (PNS). They resulted in various decoy state strategies^11,12^, which seem to help providing protection against PNS by the cost of monitoring multiple additional channel statistics besides the simple loss. To the best of our understanding, these strategies lack simple security grounds and fail to show clear and universal security proof. In general, this approach just admits the faulty use of WCPs in BB84-like protocols and tries to make up additional measures to save these (impractical) protocols, instead of finding a universal solution.

A better idea is to design new protocols, where WCP nature of information carriers is already accounted for. A valid approach known from the early days is the B92 with a strong phase reference^13^ where the presence of the strong reference pulse makes it impossible for Eve to send vacuum states if the USD fails to get a conclusive result. Another alternative first coined in^14^ with single photons and later re-invented in a practical form in^15^ is to use relativistic limitations. They allow to force Eve make decisions about her actions *before* she can actually measure the state in the line, thus breaking her only winning strategy due to causality. In this paper we demonstrate an improved experimental realization of this protocol, where we implemented an efficient one-way configuration with an active single-mode free-space channel tracking system, demonstrating stable operation over 180 m.

## Results

**Figure 1:**
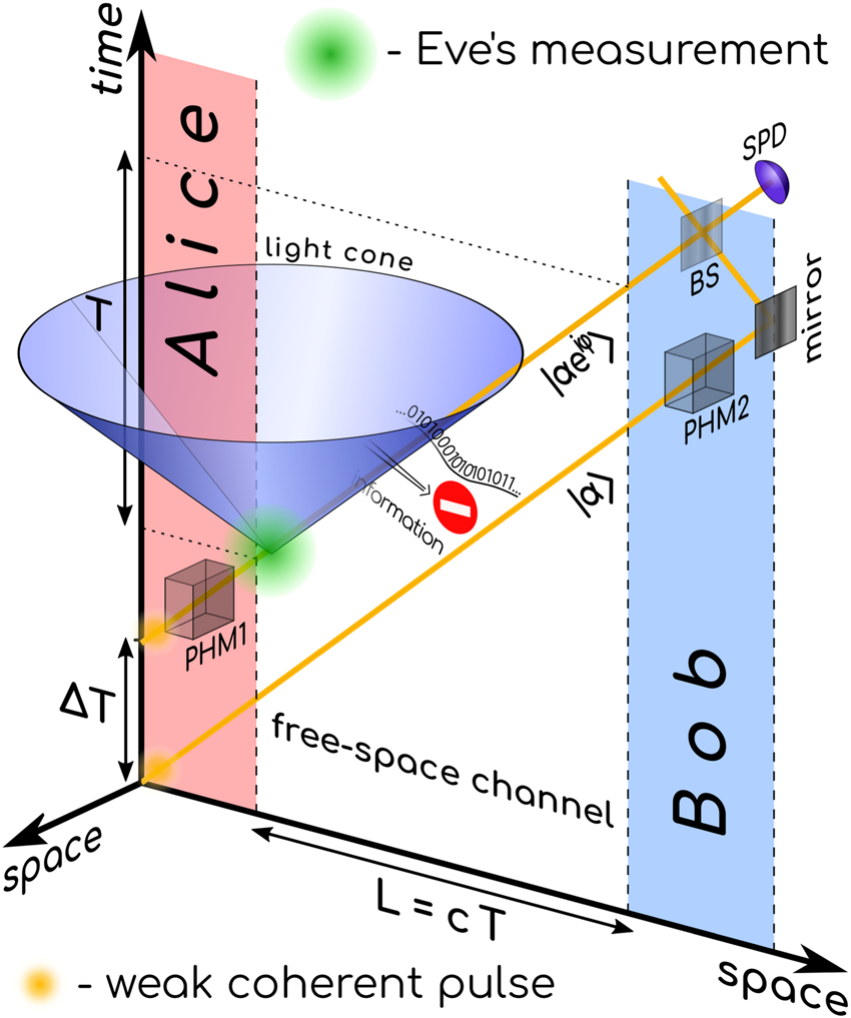
Space-time diagram of the relativistic protocol. The two pulses travel with the speed of light, thus, forbidding Eve’s actions on the first pulse dependent on her measurement of the second, modulated one. PHM–phase modulator, BS–symmetric beamsplitter, SPD–single-photon detector.

The relativistic protocol is schematically shown in Fig. 1 as a space-time diagram. Its key component is the quantum transmission with the speed of light in two time windows separated by a measurable time interval Δ*T*. The key generation procedure looks very much like B92 protocol^13^. To establish one bit of the raw key Alice and Bob randomly choose one bit of information each: *b*_*A*_ and *b*_*B*_ respectively, where *b* ∈ {0, 1}. Alice transmits two pulses: a reference WCP *α* in the first time window and a signal $|{e}^{i{b}_{A}\phi }\alpha \rangle$ in the second. Bob applies a phase shift of *b*_*B*_*φ* to the first time window and measures the result of interference between the two. He can only detect a photon if *b*_*A*_ ≠ *b*_*B*_, otherwise there is a destructive interference between the two pulses and, therefore, the vacuum state in the detector. So any time Bob’s detector clicks, he tells this to Alice and they end up with one more bit of the raw key.

In the conventional B92, eavesdropping strategy is straightforward. There is a certain probability of USD between *α* and *e*^*iφ*^*α*, so whenever Eve succeeds in her measurement she retransmits the correct state. If the USD fails Eve blocks both pulses, so the overall effect is indistinguishable from the genuine lossy channel.

The *relativistic* protocol ensures that at any moment the first pulse lies outside the light cone generated by the second one, so there cannot be any causal connection from the second to the first one. Therefore, any Eve’s measurements of the data pulse cannot affect her actions on the reference. To ensure the proper space-time relation between the pulses, the distance *L* between Alice and Bob should be known a-priori as it is a critical security parameter of the protocol. All signals delayed in the channel by more than *L*/*c*, where *c* is the speed of light, are ignored. In this modified framework Eve has no ability to block the reference pulse depending on her measurement result, as it would contradict the causality principles. However, if any one of the two pulses in the channel is missing, Bob sees the results uncorrelated with the states Alice sent, producing errors in the raw key. So whenever Eve lets the reference through, but *then* fails to measure the data pulse, she causes errors in the raw key. On the contrary, if she blocks the reference, but retransmits even the correct data pulse, she causes errors too. This picture has much in common with the strong phase reverence version of B92. The strong classical reference cannot be removed from the channel because this is directly detectable. If, on the contrary, it is present without the correct WCP companion, it inevitably produces errors. While both approaches offer ultimate protection against USD, we believe that our relativistic protocol is less technology demanding and can be more practical.

Each detector click gives Bob one bit of information, as he effectively performs post selection, i.e. chooses only those pulses for which his measurement succeeds. On the contrary, Eve’s actions cannot depend on her measurement results, otherwise Bob would see uncorrelated with Alice bits. When Eve gets a measurement result, i.e. not earlier than the second time window, it is already too late to reach the first window, located beyond the light cone^16^. Therefore, her information per any channel use is fundamentally limited by the capacity of such binary quantum channel, i.e. by the Holevo quantity^17^. The difference between Bob’s information and the fundamentally limited information of Eve gives the room for secret key generation. That is, transmission with the speed of light over the known distance, together with precise synchronization and timing, gives a new security component to QKD that offers assured protection against USD-based and any intercept and resend attacks.

An efficient experimental realization is another question addressed in this work. Transitioning to a one-way quantum channel configuration makes the system more protected from Eve’s actions, compared to the double-pass one^15^, where Eve could manipulate classical pulses traveling from Bob to Alice. It also greatly improved the operation rate of the system, as there is no need to wait the round-trip time to send more data into the channel.

As the security of such causality-based *relativistic* protocol relies on precise timing, synchronization plays a critical role in the protocol. Malicious altering of the synchronization process may easily break the foundations of the protocol security, opening a backdoor for eavesdropping. Therefore a special secure procedure was developed to guarantee proper synchronization during the protocol operation. It requires a backward classical channel where information travels with the speed of light.

To initiate quantum transmission Bob generates a random bit sequence and sends it to Alice using the classical channel with the same rate that Alice uses for QKD. At each received bit Alice stores it in her local memory and transmits one WCP into the quantum channel. After the whole packet is transmitted, Bob and Alice compare their synchronization sequences. If the sequences are the same, Alice can guarantee that she received each bit not earlier than Bob expects her to get it. Otherwise, it would be a superluminal information transfer between Bob and Alice, which contradicts the relativity theory. That directly means that Alice never sent any quantum state into the channel earlier than Bob thinks she did. At the same time, this is the only case when Eve would have an extra time to act *after* her measurement without causing errors: if she could force Alice to transmit earlier than Bob thinks, the protocol would be broken. If, on the contrary, Alice sends her pulses later, Bob just will not receive any correlated with Alice raw key, so the packet will be discarded as not containing any secret information. If after comparison the synchronization sequence received by Alice appears to differ from that of Bob, it is a potential sign of an ongoing synchronization attack and the whole packet must be discarded as unreliable.

The backward communication channel required for synchronization is realized via the tracking system, which also serves for transmission of service data and control messages in both directions between the parties. Besides data communication, the tracking system is needed to keep the quantum channel up, as, in the contrast to conventional free-space QKD systems, the present one needs a single mode receiver, which is compatible with a fiber-based delay interferometer. Without active tracking, the system was extremely unstable when mounted on standard theodolite tripods and would not operate reliably even for a few minutes. With the tracking system implemented it showed good performance at least for hours, although we did not check the stability for a longer time. More detailed information about the single mode channel and the tracking system can be found in Supplementary. There is also a discussion about the difference between the group velocity of pulses in the air and the speed of light, which is insignificant for the implemented parameters of the protocol.

**Figure 2:**
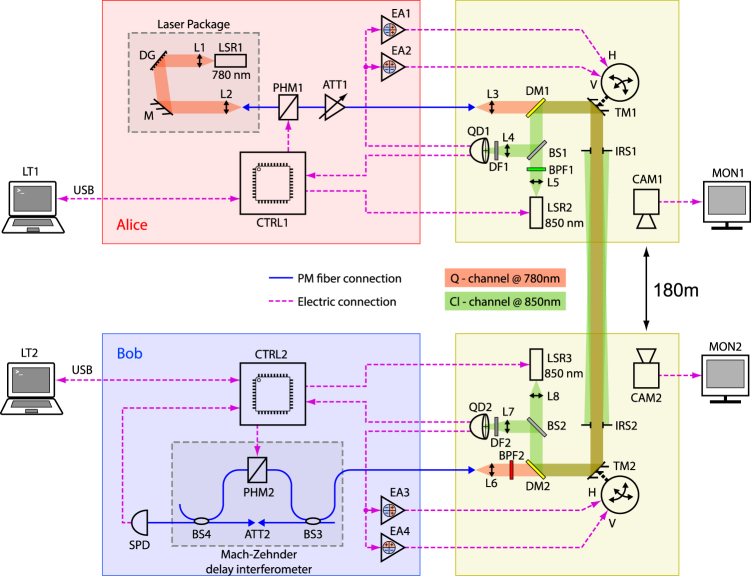
Experimental setup schematic. LT–laptop-based user station; DG–diffraction grating in Littrow configuration; L–lens; M–mirror; PHM–150 MHz lithium niobate fiber-coupled phase modulator; ATT–variable optical attenuator; CTRL–control electronics; EA–electronic error amplifier in the tracking feedback loop; DM–dichroic mirror; TM–piezo tip-tilt mirror; QD–quadrant photodetector; DF–ground glass-based diffuser; BS–symmetric beamsplitter; IRS–25 mm iris diaphragm; BPF–band-pass filter; CAM–coarse pointing camera; MON–user monitor for the camera; SPD–silicon avalanche photodiode-based single-photon detector.

**Figure 3:**
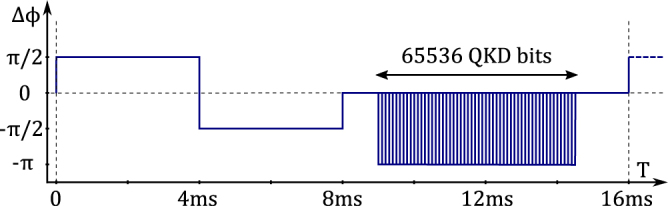
Operation of the receiving interferometer phase modulator. In each 16 ms cycle first it measures count frequencies in two quadrature points to adjust the bias, and then proceeds to the QKD sequence.

Another experimental challenge addressed in our one-way design is the proper alignment of the receiving side interferometer. To simplify the setup we eliminated the transmission side delay interferometer altogether and used a CW laser instead. Thus, Alice’s side contains only a narrow linewidth CW laser (external cavity diode laser), a phase modulator and an attenuator, as shown in Fig. 2. The receiving side has a polarization maintaining fiber based delay interferometer with a phase modulator in one of the arms, which serves for both interferometer alignment (with a quasi-DC bias) and data modulation during the QKD stage. The bias is constantly adjusted according to the number of single photon detector clicks when biased at *π*/2 below and above the normal level that corresponds to the dark interferometer output. A whole cycle of the modulator work is shown in Fig. 3. More details on interferometer alignment are found in Supplementary.

The main operation parameters are as follows. Each transmitted quantum symbol is a 10 ns long piece of the CW laser signal at *λ* = 780 nm with the output intensity of −92.9 ... −78.9 dBm, which corresponds to 0.02 ... 0.5 photons per pulse. The delay Δ*T* in the receiving interferometer is 20 ns, so each symbol interferes with the corresponding chunk of the CW signal going Δ*T* ahead (the phase reference window). The depth of phase modulation equals 0.8*π*. Phase modulated symbols come in packets of 65536 bits each with the average rate of 25 MHz. A packet can be sent in any phase modulator cycle, which is 16 ms long (see Fig. 3). However, the actual packet rate was limited by the time needed to exchange the random data buffers and measurement results with a PC via a USB interface, so the actual rate was about 2 packets/sec.

**Figure 4:**
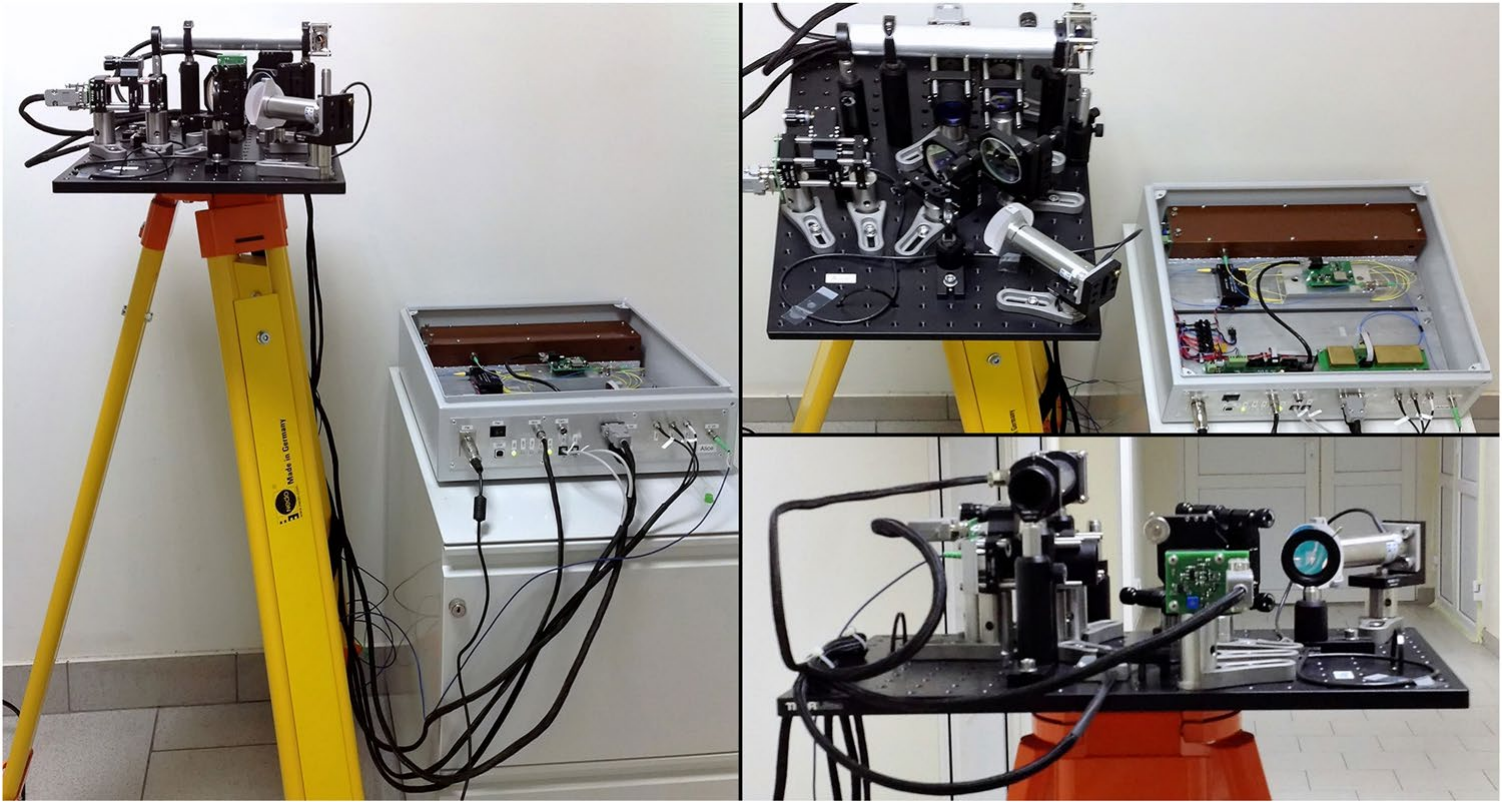
Station Alice: a tripod with a free-space channel tracking platform and a box with fiber optic components and all electronics.

The whole system consists of two similar stations, each containing a box with electronics and fiber-based elements, and a free-space channel tracking platform placed on a tripod as shown in Fig. 4. The quantum single-mode free-space channel uses diffraction-limited 1″ diameter aspheric lenses to collimate radiation to/from single-mode polarization maintaining fibers. Quantum signals are spatially mixed with the 850 nm beacon radiation used by the tracking system. Beacon light is detected by a quadrant photodiode to provide feedback to the piezo driven steering mirror. It also delivers a 25 Mbit/s Manchester encoded classical signal used for secure synchronization and transfer of auxiliary information between stations. The tested channel length of 180 m was actually limited by the length of the building, while the system itself was designed to operate over as far as 400 m.

**Figure 5:**
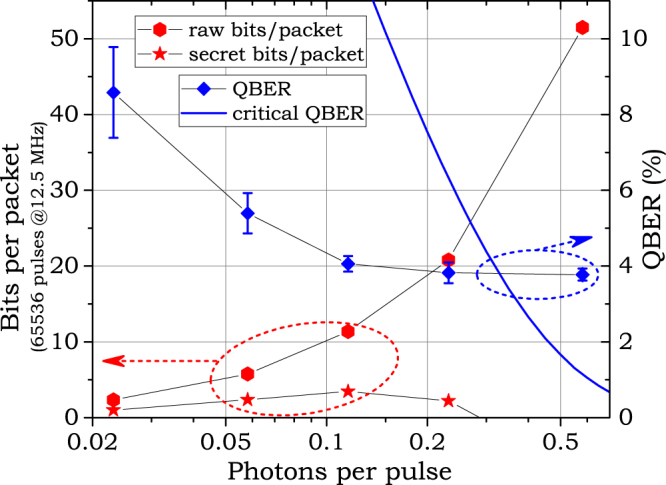
System efficiency and QBER measured in PRBS operation mode vs. the average photon number. The figure also shows the calculated number of asymptotic secret bits per packet as well as the critical QBER, above which no secret bits can be extracted. Error bars on the QBER plot are purely statistical ones corresponding to the uncertainty of QBER estimation based on the finite number of obtained bits. More precisely, they depict a 95% binomial proportion confidence interval for all raw bits accumulated in a particular setting.

The system operates in two modes: with pseudo-random bit sequences (PRBS) and with real random data. The first one is used for testing purposes as it provides an easy way to calculate quantum bit error ratio (QBER) without utilization of the classical channel (stations know the pseudo-random sequences used at the other end of the line). The second mode works with real random data from a quantum random number generator (QRNG)^18^ stored at laptops. Figure 5 shows system efficiency and QBER for the PRBS operation mode at different photon levels.

To estimate the asymptotic secret key rate we use the information based approach. The raw information obtained by Bob must be reduced to eliminate the Eve’s information, or more accurately, the information that could potentially leak to Eve. As the raw key always contains some errors, a portion of the raw key is also used for error correction. As discussed earlier, the implemented relativistic scheme disallow Eve’s influence on the received quanta in the way that her actions depend on results of her measurements. Without this ability to post-select, Eve cannot decide which pulses will travel to Bob and produce detector clicks and which she will block contributing to the channel loss. At most she can obtain the average information per pulse. Effectively, Eve’s information is bounded by the Holevo quantity^17^:

$$\chi (\mu ,\phi )=h(\frac{1-\exp [-2\,\mu \,{\sin }^{2}(\phi /2)]}{2}),$$

where *h*(*p*) = −*p*log(*p*) − (1 − *p*)log(1 − p); *φ* = 0.8*π* is the modulation depth, and *μ* is the average number of photons per pulse. Ideal asymptotic error correction requires *h*(*QBER*) bits, so the overall asymptotic secret key rate equals **R** = 1 − χ(*μ*, *φ*) − *h*(*QBER*). It should be noted that here we do not take into account any finite-size effects, as they do not qualitatively change the results. Some elaboration for finite-sized sequences is published elsewhere^16^.

At small *μ* Eve’s information is small, but the estimated secret key length is severely limited by the high QBER. At large *μ* QBER decreases, however, the Eve’s information becomes the limiting factor. The maximum efficiency is observed at around *μ* = 0.1 as follows from Fig. 5.

**Figure 6:**
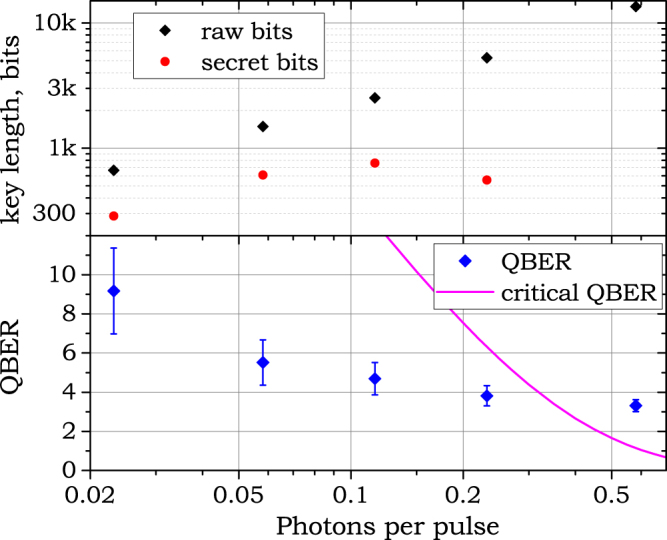
Key lengths and QBERs vs. the average photon number for QKD with random data from the QRNG. Each point is a result of QKD with 16 Mbit input buffers, i.e. 256 packets transmitted. Error bars on the QBER plot show the 95% binomial proportion confidence interval for the particular raw key obtained in the corresponding data point.

Operation with real data from QRNG was performed to distribute actual raw keys. Privacy amplification and error correction was not implemented in the experiment, as it is relatively straightforward, but too time consuming for this proof of principle demonstration. Therefore, all estimations are made using the asymptotic relation found above and the obtained raw keys. Figure 6 shows experimentally measured data—raw key length and QBER—as well as asymptotically estimated number of secret bits. Each data point shows the result of a particular exchange of 1.68 × 10^7^ WCPs between Alice and Bob. The most efficient secret key generation was observed at *μ* = 0.116, where the raw key generation rate (inside a packet) equals 2170 bits/sec and the asymptotic secret key rate is estimated as 660 bits/sec. As mentioned earlier, average rates are substantially smaller due to the slow data exchange with laptops: 20 and 6.2 bits/sec respectively.

The performance of the single-mode free-space channel is another thing to mention. Although it was inside a building, heating and ventilation caused a significant mode distortion. Typical wandering frequency is measured to be below 10 Hz, so the tracking system with the bandwidth of 10’s of Hz substantially helped in reducing the loss. Nevertheless, active tracking could only compensate for the beam shift as a whole, but not the mode distortion. The measured free-space quantum channel loss (the ratio between the transmitted power and the Rx fiber-coupled power) is around 13 dB. At the same time the overall system efficiency, i.e. the ratio of detected photons to the transmitted ones, was 1.5 × 10^−3^.

## Discussion

The presented concept of the *relativistic* or causality-based QKD provides a new dimension to conventional quantum cryptography. Its main advantage is in complete decoupling between the channel loss and the security level. No additional tests are required (at least in theory), besides the standard privacy amplification and error correction, to guarantee information theoretic key security. In this sense it has much in common with the original B92 protocol with strong reference pulses. At the same time, the presented protocol seems to be less technology demanding, as the former requires extremely high contrast ratio between the signal and the reference. Brief estimation shows that at a typical system efficiency of 10^−3^ and the average signal photon number of 0.1, reference pulses should be as bright as 10^4^ photons per pulse to be reliably detected by the receiver, i.e. 10^5^ times the power of signal pulses. It is very experimentally challenging to maintain reliable interference between the two, as most optical elements, such as beamsplitters, have a typical contrast of at most 10^3^ due to parasitic reflections and scattering. This is probably the main reason why, to the best of our knowledge, there is no experimental demonstration of the original B92 yet. The relative simplicity of the presented protocol, however, comes in exchange for the additional assumptions that we need from the channel, namely, the knowledge of the channel length.

The channel length or, more precisely, *distance* between Alice and Bob plays a critical security role in the relativistic protocol. It is an important security parameter, which should be known a-priori to guarantee the protocol security. Formally, one cannot be more confident in the security of the generated keys, than he is confident in the distance between the parties. However, this can be eased by placing a restriction only on the lower bound of the channel length.

In fact, increasing the delay Δ*T* between two pulses one can tolerate more deviation between the actual time of flight and *L/c*. There is a more detailed discussion on that subject in Supplementary, but in general one has to make sure that the second pulse cannot overtake the first one even if the second one travels along the straight line between Alice and Bob with the speed of light. Thus, the minimal required delay between the pulses equals Δ*T*_min_ = 2(*T*_*o*_ − *L*_min_/*c*), where *T*_*o*_ is the *observed* time of flight, *L*_min_ is the lower confidence bound for the value of *L*, and the factor of 2 is included because in this particular implementation the synchronization process relies upon the same channel and therefore can be offset by the same amount. It could be, however, cut in half if an external trusted synchronization scheme is used.

As *L* is always positive, Δ*T* > 2*T*_*o*_ is a safe, but often impractical choice. To remain practical, one would want Δ*T* ≪ *T*_*o*_. This is feasible for a large-distance free space communication with a moving target confined in some relatively small area, e.g. inside a town. Another possible strategy is using hollow core photonic crystal fibers (PCFs), where the effective refraction index is demonstrated to be as low as 1.003 and the optical loss is expected to beat that of conventional silica fibers^19^. Future hollow core PCF infrastructure may become the natural backbone for the relativistic QKD network, since the difference between the propagation speed and *c* is minimal in such fibers.

Yet another practical possibility is to use the same phase-encoding hardware either in conventional (when no reliable information about distance is available) or relativistic mode. This may be a good compromise for attaining the best possible security scenario depending on the particular circumstances.

In conclusion, we report a *relativistic* QKD system, which, unlike conventional protocols, offers inherent resistance against USD based attacks under arbitrary large channel loss while using practical weak coherent pulses as information carriers. Our experimental setup operates via a 180 m uni-directional single-mode free-space quantum channel with the active tracking system. Due to its simple structure and straightforward security foundations, this protocol may become the first *practical* QKD protocol with as general a security proof as for BB84. Its advantages are best attained in line-of-sight metropolitan links up to several kilometers long between stationary objects or in the future low loss hollow core PCF networks, where ultimate security needs meet the ease of experimental realization.

## Methods

### Hardware implementation

The light source is a CW-driven 90 mW 780 nm laser diode with an external cavity based on a 1800/mm diffraction grating in Littrow configuration. Phase modulators used are low frequency PM fiber coupled lithium niobate modulators that have no internal electrical waveguide and termination. Unlike traveling wave modulators, this type can be used for simultaneous interferometer adjustment and high-speed phase modulation due to their tolerance to large DC offsets. The single photon detector is based on a silicon Geiger mode avalanche photodiode package with the internal thermoelectric cooler. Its quantum efficiency is 35% and the dark count rate is around 700 Hz. The tracking system uses PI S-330.80 L piezo tip-tilt platforms with 2″ mirrors. The main resonance frequency for this steering mirror configuration is around 920 Hz. As a beacon light source we use a 10 mW directly modulated 850 nm laser diode. Its radiation is collimated using 0.5NA *F* = 8 mm aspheric lens. Quadrant photodiodes have 3 × 3 mm^2^ active area and they are placed in the focal plane of the *F* = 80 mm focusing lens. To smooth the feedback response a 1500 grit ground glass diffuser is placed a few mm before the photodiode. The AC component of the detected signal is summed from all the quadrants, is frequency corrected, amplified and converted to a binary data stream–the classical communication channel. The DC component is amplified separately for all the quadrants and then the vertical and horizontal error channels are formed by pairwise subtraction of corresponding signals. The error signals are scaled with respect to the total received power and are input into the two PID control loops. Fine synchronization between the stations is performed by the PLL which locks to the received digital waveform of the classical channel. The used Manchester encoding ensures that there is enough zero crossings for the PLL to operate regardless of the transmitted data.

## Electronic supplementary material

Supplementary information
