# Supplementary material for: Relativistic quantum key distribution system with one-way quantum communication
Source: Sci Rep. 2018 Apr 17;8:6102. doi: 10.1038/s41598-018-24533-6 (PMC5904211; doi:10.1038/s41598-018-24533-6)
Supplement: Supplementary file 1 — Supplementary information [file 41598_2018_24533_MOESM1_ESM.pdf]

Relativistic quantum key distribution system with one-way quantum communication

K. S. Kravtsov[1,2,*], I. V. Radchenko[1,2], S. P. Kulik[1,3], and S. N. Molotkov[4,5,6]

[1] *Quantum Technology Centre of Moscow State University, Moscow, Russia*

[2] *A.M. Prokhorov General Physics Institute RAS, Moscow, Russia*

[3] *Faculty of Physics, Moscow State University, Moscow, Russia*

[4] *Academy of Cryptography, Moscow, Russia*

[5] *Institute of Solid State Physics, Chernogolovka, Moscow Rgn., Russia*

[6] *Faculty of Computational Mathematics and Cybernetics, Moscow State University, Moscow Russia*

[*] *ks.kravtsov@gmail.com*

(Dated: April 9, 2018)

SUPPLEMENTARY MATERIALS

System operation and data flow patterns

Figure S1 shows the data flow chart within the system. Before the protocol starts, Alice and Bob obtain random sequences from the laptops and store them in buffers PHM_A, PHM_B, and SYNC_B. When both stations are ready, Bob initiates the transfer by sending a request code. Alice replies with an acknowledgment to indicate that she is also ready to proceed to QKD. After the acknowledgment is received, Bob begins transmission of the synchronization sequence. For each bit received Alice replies with a quantum state, modulated according with the PHM_A value. She also stores the received synchronization bit into SYNC_A. Bob uses PHM_B data to change the state of the receiving interferometer and stores single-photon detector clicks into SPD_B.

After the packet is transferred Alice copies SYNC_A buffer to her laptop, and Bob does the same with the SPD_B buffer. The rest is performed using the laptops with TCP/IP connection between them. First, Alice and Bob compare the contents of their synchronization buffers: SYNC_A and SYNC_B. If they differ, the packet is discarded and is not used as a raw key. If they are the same, the key sifting is performed. Bob tells Alice the positions in SPD_B when his detector produced clicks. Alice creates her raw key from her PHM_A data at the specified positions. Bob uses PHM_B for the same purpose, but inverts all the data. Ideally, Alice and Bob should arrive to the same key. However, experimental imperfections and dark detector counts lead to errors. For the purpose of current publications the raw keys were directly compared to calculate corresponding QBER and estimate the asymptotic secret key rate.

Relativistic protocol and the presence of air in the channel

An important question is whether the protocol remain secure under the presence of air in the quantum channel. So far in the model we assumed that all signals in the quantum channel propagate with the (vacuum) speed of light, which is not the case for terrestrial line-of-sight atmospheric links. The answer directly depends on the channel length and the delay ΔT between the two WCPs in the quantum channel. If ΔT cannot be compensated during the round trip by the eavesdropper substituting a vacuum channel instead of the atmospheric one, the system remains perfectly secure, as all the assumptions remain correct. Theoretically, increasing ΔT we can achieve secure operation even in the case of an optical fiber based link. However, this becomes largely impractical as the required delay equals a significant fraction of the communication distance. So the receiving delay interferometer needs to be almost as large and lossy as the channel itself, which is undesirable. Going to the extreme, any channel type can be supported if one can guarantee that the first WCP reaches Bob's setup *before* the second one leaves Alice's. This situation has much in common with [S1], where this sequential quantum transfer was proposed for the first time, but with single photons.

For calculations we assume that the air refraction index is 1.0002804 that corresponds to the group velocity in dry air at 15 °C, 101.325 kPa and with 450 ppm CO_2 content at the wavelength of 780 nm. The maximal channel length is given by

$$L_{\max} = \frac{1}{2}\frac{c\Delta T}{n-1},$$

which gives $L_{max} = 10.7$ km at $\Delta T = 20$ ns. Therefore, current experimental realization is well within the maximum range limited by the presence of air, so it is as secure as it would be with the vacuum quantum channel.

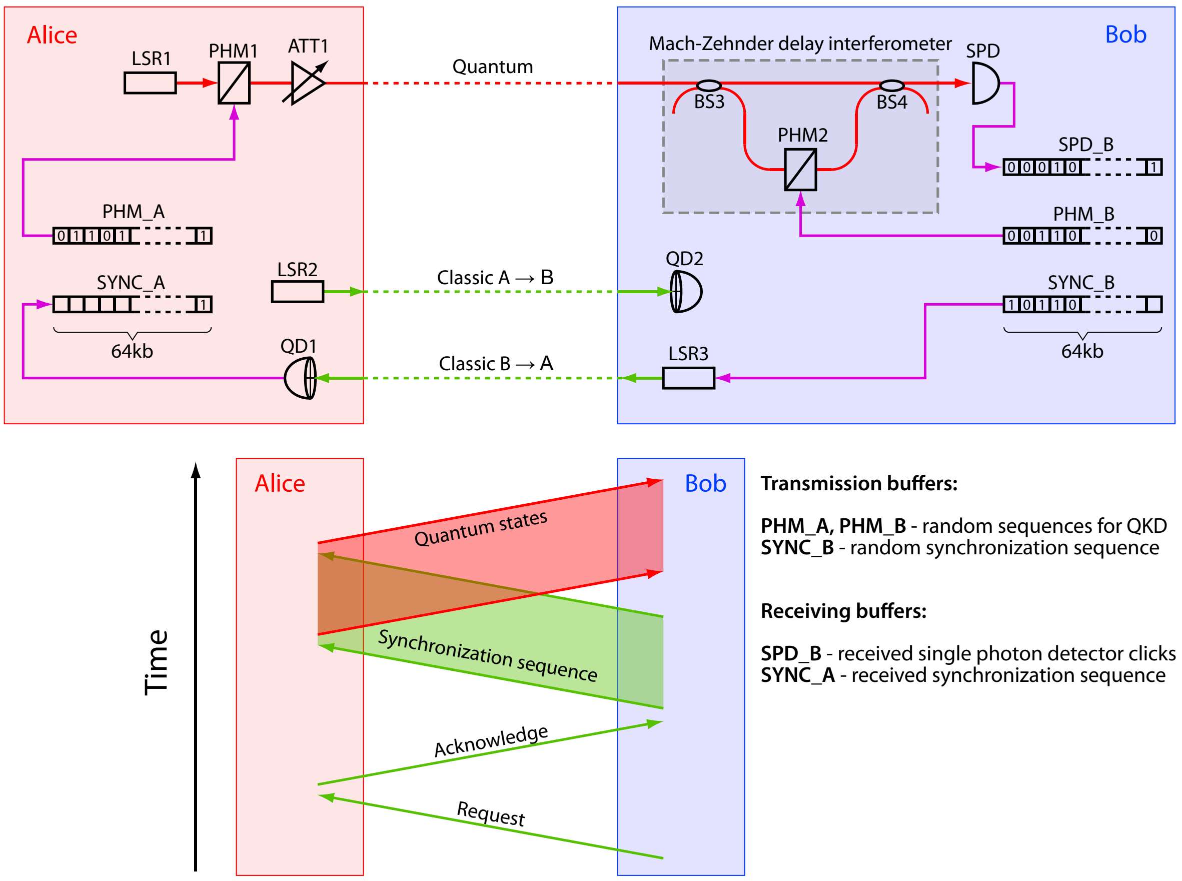

FIG. S1. Data flow chart during system operation.

Receiving side interferometer alignment

In order to operate properly, the receiving side delay interferometer must be aligned such that without any phase shifts no light propagates into the single-photon detector (SPD). In practice, the required phase shift is constantly changing due to thermal variations of the optical path lengths and also due to slow wavelength drifts. Typical time-scale of these variations is in the order of a minute or even shorter.

To solve the problem we implemented a closed loop control that takes the error signal from the SPD measurements and adjusts the bias voltage. The error signal is a normalized difference between the number of detector counts when biased above and below the current estimate, see Fig. S2. The smaller the number of counts the larger are the statistical fluctuations of the error signal, so the obtained error signal is scaled appropriately to ensure stable convergence to the best estimate.

The system uses two 4 ms long time windows to calculate detector counts when biased below and above the current value. The rest of the 16 ms time frame is used for quantum key distribution. Then all the steps are repeated again. The effective feedback speed depends on the signal strength and usually is at least several Hz, which is enough to track the phase changes in real time.

The phase modulator can produce phase shifts of several wavelengths, but nevertheless sometimes the bias voltage needs to keep increasing even when it hits its allowed maximum value. In this case our digital feedback scheme makes a step back by several full wavelengths, decreasing the required voltage but keeping the same phase relations. The described scheme proved to work well under broad range of conditions, and can be easily relied upon in practice.

Single mode free-space channel and tracking system

The single mode free-space channel itself is an advanced piece of equipment. The presence of moving air in the channel makes the link to have dynamic behavior and, therefore, requires an active tracking system.

The active tracking system corrects only for the most critical channel disturbance — deviation of the beam from a straight line. Higher order perturbations, not accounted in our approach, lead to beam profile distortion, which also contributes to the coupling loss, but requires much more advanced adaptive optics tools to cor-

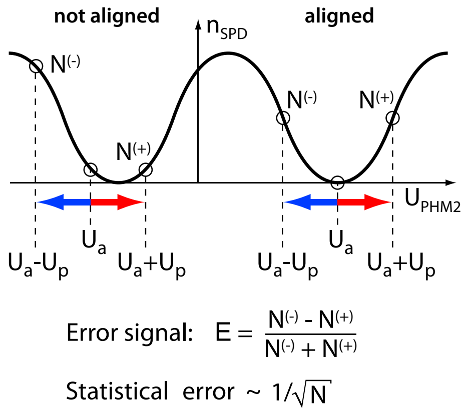

FIG. S2. Receiving side interferometer transmission vs. phase modulator bias voltage. If the interferometer is not properly aligned (left side), equal bias voltage offsets produce unequal numbers of detector clicks, which contribute to the error signal correcting U_a. If it is already aligned (right side) error signal will be zero except for the statistical deviations due to the limited number of detector clicks, which are averaged out with time. No U_a correction is carried out in this case.

rect. Previous reports [S2, S3] show satisfactory behavior of similar tracking systems at operation distances up to 1 km at telecommunication wavelengths. This gives us confidence that such systems can be extended to even longer metropolitan scale free-space single mode links, that makes our *relativistic* QKD approach viable and ready to substitute some conventional fiber-based QKD methods. Another experiment [S4] confirms that even a 150 km range is feasible for a single mode free-space link. As the security of the proposed QKD protocol is totally decoupled from the channel loss, future development of low dark count nanowire single photon detectors may overcome current system loss limitation and make it suitable for 100 km range free-space QKD.

Operation of our tracking system does not require any classical communication channel between the stations. Each station performs the following task: it measures the direction of the beacon light arrival and points the transmission beam exactly at the same direction. Due to the link reciprocity, this makes sure that the transmitted beam always reaches the destination. In other words, each station has its own closed loop control of the steering mirror, and this is enough for a stable operation of the whole link.

Beacon light is slightly defocused such that it creates a half meter diameter spot at the end of the channel. Initial setup requires coarse pointing of the stations to each other, so each of them could see the beacon light from the other. After this is achieved, stations fall into the closed loop control mode and the quantum link becomes ready for QKD. Importantly, after link downing when something blocks the beams, it reliably recovers by itself and does not need any operator intervention.

[S1] L. Goldenberg and L. Vaidman, "Quantum cryptography based on orthogonal states," *Phys. Rev. Lett.*, vol. 75, no. 7, pp. 1239–1243, 1995.

[S2] Y. Arimoto, "Multi-gigabit free-space laser communications using compact optical terminal with bidirectional beacon tracking," in *IEEE International Conference on Communications, (ICC 2007)*, Kyoto, Japan, Jun. 2007.

[S3] ——, "Operational condition for direct single-mode-fiber coupled free-space optical terminal under strong atmospheric turbulence," *Opt. Engineering*, vol. 51, no. 3, p. 031203, 2012.

[S4] D. W. Young, J. E. Sluz, J. C. Juarez, M. B. Airola, R. M. Sova, H. Hurt, M. Northcott, J. Phillips, A. McClaren, D. Driver, D. Abelson, and J. Foshee, "Demonstration of high data rate wavelength division multiplexed transmission over a 150 km free space optical link," in *Military Communications Conference, (MILCOM 2007)*, Orlando, FL, USA, Oct. 2007.
